# Understanding the scope of intracardiac echocardiography in catheter ablation of ventricular arrhythmia

**DOI:** 10.3389/fcvm.2022.1037176

**Published:** 2022-10-28

**Authors:** Chuanzhen Ma, Tongshuai Chen, Yanbo Chen, Junye Ge, Wenqiang Han, Qinhong Wang, Jingquan Zhong

**Affiliations:** ^1^The Key Laboratory of Cardiovascular Remodeling and Function Research, Chinese Ministry of Education, Chinese National Health Commission and Chinese Academy of Medical Sciences, The State and Shandong Province Joint Key Laboratory of Translational Cardiovascular Medicine, Department of Cardiology, Qilu Hospital, Cheeloo College of Medicine, Shandong University, Jinan, China; ^2^Department of Cardiology, Qilu Hospital (Qingdao), Cheeloo College of Medicine, Shandong University, Qingdao, China; ^3^Department of Cardiology, Weifang People’s Hospital, Weifang, China

**Keywords:** intracardiac echocardiography, ventricular arrhythmia, catheter ablation, 3D mapping system, cardiac structures

## Abstract

Over the last few decades, catheter ablation has emerged as the first-line treatment for ventricular arrhythmias. However, detailed knowledge of cardiac anatomy during the surgery remains the prerequisite for successful ablation. Intracardiac echocardiography (ICE) is a unique imaging technique, which provides real-time visualization of cardiac structures, and is superior to other imaging modalities in terms of precise display of cardiac tissue characteristics as well as the orientation of anatomical landmarks. This article aimed to introduce the various advantages and limitations of ICE in the ablation of ventricular arrhythmias.

## Introduction

Timely intervention is needed for ventricular arrhythmias (VAs) that cause palpitation, chest tightness and other symptoms, as well as that can lead to or have led to cardiomyopathy. Necessary therapeutic measures for VAs usually consist of specific drug therapy like beta-blockers (β-blockers) and class Ic antiarrhythmic drugs. However, in cases of ineffective drug therapy or inadequate patient compliance, catheter ablation is considered to be an effective means of treatment (1). Intracardiac echocardiography (ICE) refers to the placement of an ultrasound probe at the catheter tip, which is then transported to the cardiac cavity through peripheral blood vessels. Hence, this process enables precise cardiac anatomy visualization without air and other interference factors, thereby providing optimum outcomes. It provides high-resolution real-time visualization of cardiac anatomy as well as the catheter placement, thereby dynamically monitoring the entire ablation process. ICE also helps in understanding the spatial relationship between mapping the ablation catheter and corresponding cardiac structures and guides the degree of attachment between the top of the ablation catheter and corresponding anatomical landmarks (2). It can easily monitor the formation, location, extent, and degree of ablation injuries for assessing the safety and efficacy of ablation. Additionally, ICE also continuously monitors complications as well as determines their location and severity in real-time, such as pericardial effusion (3–5) or thrombosis (6, 7). Based on this, procedural complications can often be detected and treated in time before the hemodynamic changes occur (2). Moreover, ICE can also display all cardiac structures and accurately locate the aortic root and the pulmonary sinus by operating in the right cardiac system. The ablation of outflow tract is more instructive, and it is of greater significance for mapping and ablation of related arrhythmias originating from the structures protruding from the cardiac cavity, such as papillary muscle (PM), false tendons (FT), and moderator band. Simultaneously, it also reveals the functional changes in myocardial echo and thickness (8, 9) and helps in the accurate localization of substrates such as scars. Other benefits of this technique include excellent patient tolerance, low radiation and contrast agent exposure, and lack of need for general anesthesia (2).

## Classification of intracardiac echocardiography systems

Common ICE systems are divided into the following two types:

(1) *Radial or rotating ICE:* This system uses a single piezoelectric crystal attached to the tip of a 6-to 10-French catheter. The rotating sensor operates at an imaging frequency of 9–12 Mhz and provides images within 6–8 cm around the probe during surgery. The resultant images are omnidirectional tomography images perpendicular to the long axis of the catheter and similar to intravascular ultrasound images.

(2) *Phased-array ICE:* It consists of a 64-element ultrasonic probe crystal mounted on the distal end of an 8-to 10-French catheter which provides a perpendicular sector view. As the ultrasonic frequency of the probe is variable (5–10 Mhz), it can bend in four directions: anterior (A), posterior (P), left (L), and right (R), with a maximum bend angle up to 160°. This catheter displays 2D pulse/Doppler ultrasound imaging with a depth up to 15–16 cm. Phased-array ICE possesses several advantages that include greater radial depth, Doppler imaging ability, and higher operability. CARTOSOUND software is a new imaging technology that integrates ICE with 3D electro anatomical mapping systems. It consists of a magnetic sensor embedded within the phased-array ICE catheter tip that allows integration of 2D ultrasonic images developed by intracardiac ultrasonic catheter with 3D magnetic field information obtained by the 3D electro anatomical mapping system. Hence, this technology is the cumulative, precise combination of 3D magnetic field positioning and navigation along with real-time 2D ultrasonic technology.

## The standard intracardiac echocardiography view

**FIGURE 1:**
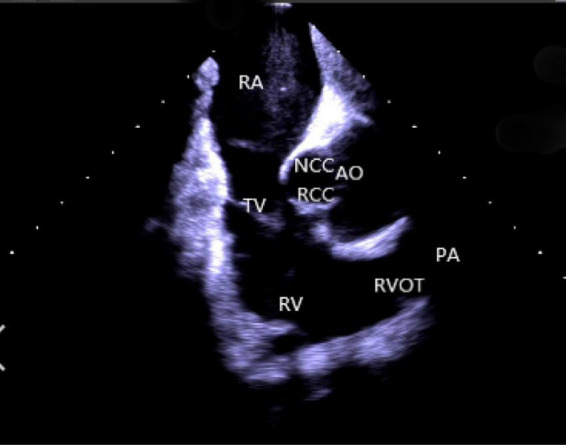
Homeview position. RA, right atrium; TV, tricuspid valve; RV, right ventricle; NCC, non-coronary cusp; RCC, right coronary cusp; AO, aortic valve; RVOT, right ventricular outflow tract; PA, pulmonary artery.

**FIGURE 2:**
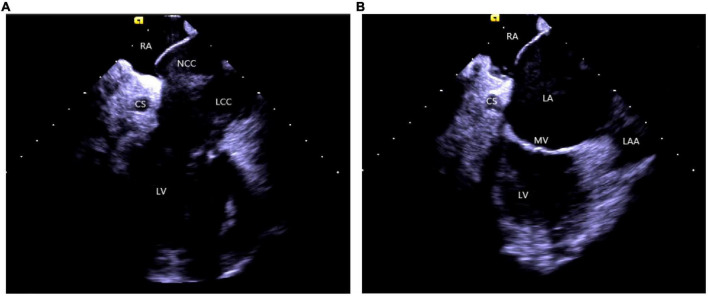
**(A)** The clockwise rotation from the HomeView position. **(B)** The clockwise rotation from the A position. RA, right atrium; CS, coronary cusp; LV, left ventricle; NCC, non-coronary cusp; LCC, left coronary cusp; RVOT, right ventricular outflow tract; LA, left atrium; MV, mitral Valve; LAA, left atrial appendage.

(1) *The introduction of ICE:* Experienced operators usually use a bilateral femoral vein approach to enter the cardiac cavity without any radiographic imaging. The basic principle is to always maintain a clear echogenic space (black) in front of the catheter and avoid pushing it when showing an echogenic space (white) (2).

(2) *Right atrium operation and view:* After positioning the ICE catheter in the mid-right atrium (RA) through the inferior vena cava, the catheter was rotated so that the ultrasound probe points to the center of the tricuspid valve (TV), thereby reaching the HomeView position. This important position provided imaging of the RA, TV, right ventricle (RV), aortic long axis, non-coronary, and right coronary cusps, as well as a small part of the right ventricle outflow tract (RVOT) (Figure 1). From the HomeView position, a clockwise rotation exhibited the RV long axis model, showing the RA, coronary sinus, non-coronary and left coronary cusps, and part of the left ventricle (LV). Further clockwise rotation identified the left atrium (LA) and displayed the LA, RA, coronary sinus, left atrial appendage, mitral valve, and LV, respectively (Figure 2).

Other commonly used views: Based on HomeView, the catheter was positioned in the high RA and was rotated clockwise slightly to display the short axis of the RVOT, pulmonary valve, and sinus, the long axis of the left ventricle outflow tract (LVOT) and the long axis of the aorta, respectively.

(3) *Right ventricular operation and view:*

**FIGURE 3:**
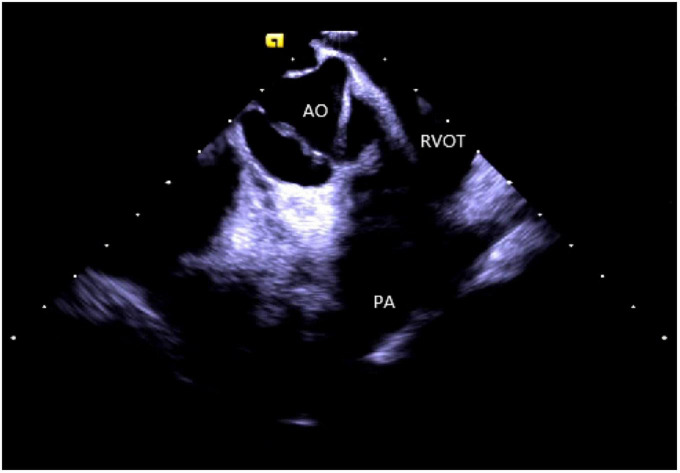
The short axis of the aorta. AO, aortic valve; RVOT, right ventricular outflow tract; PA, pulmonary artery.

**FIGURE 4:**
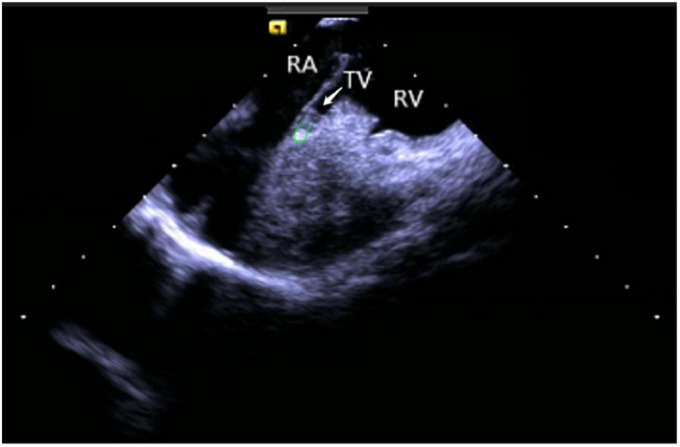
Tricuspid annulus subvalvular reflex. RA, right atrium; TV, tricuspid valve; RV, right ventricle.

Right ventricular modeling: Based on the HomeView position, the ultrasound sector rotated clockwise toward the RA posterior wall, bending the P curve and directing the catheter into the RV along with the TV orifice. With additional clockwise rotation, the RVOT came into view, along with the long axis of the pulmonary artery, the short axis of the aorta, and the aortic sinus (Figure 3). The catheter was then rotated counterclockwise toward the RV free wall and made an L-bend, thereby showing the RV, LV, moderator band, interventricular septum, and LV anterolateral papillary muscle. Additional downward movement of the catheter to the lowest apical position resulted in the loosening of the L and P bends. Then the catheter was rotated counterclockwise toward the bottom of the RV displaying the moderator band and the papillary muscle of the posterior RV. A continuous counterclockwise rotation toward the RV free wall showed the RV anterior papillary muscle, the free wall of the tricuspid annulus, the subvalvular reflex, and a part of RA (Figure 4). At this point, the RV modeling was complete.

Left ventricular modeling: Based on HomeView, the catheter was rotated clockwise to point the ultrasonic sector toward the RA posterior wall; the P curve was bent, which sent the catheter into the RV along with the tricuspid orifice. A slight clockwise rotation attached the ultrasonic catheter to the RV septum and bent the L curve to complete the modeling of the LV long axis and PM papillary muscle from top to bottom. After the completion of the LV long-axis modeling, the P and L curves were loosened, and the ultrasound catheter was rotated clockwise so that the fan pointed toward the LV bottom to complete the LV short-axis modeling.

## Application of intracardiac echocardiography in the ablation

### Application of intracardiac echocardiography in transatrial septal puncture

The common approach for conducting the ablation procedure of LV arrhythmias is usually divided into the retrograde aortic valve as well as the transseptal approaches. A transseptal approach should be chosen to avoid aortic valve injury in cases of aortic valve stenosis, dysplasia, arterial plaque formation, or children with body weight < 15 kg (10). Moreover, the transseptal approach improves the stability and attachment of the ablation catheter to the LV posterior medial papillary muscle for precise mapping and ablation (11). ICE is a valuable tool that determines the location of the oval fossa and the surrounding anatomical structures and guides transseptal puncture for accurate imaging (12, 13). During a transseptal puncture, ICE imaging from RA displays the needle tip resting on the septum, shaped like a “tent.” Once the needle passes through the septum, the “tent” collapses, revealing bright red arterial blood. Furthermore, saline injections lead to the visualization of the puncture needle as well as a “blister” sign in the LA. ICE-guided transseptal puncture is more intuitive, safe, and reliable, without any need for fluoroscopy-assisted puncture. Since ICE allows a real-time assessment of cardiac anatomy, early monitoring of the complications, like pericardial effusion or pericardial tamponade, and resolving them in time become feasible (2).

Several studies have described that transseptal puncture can be safely and effectively performed with ICE and 3D electroanatomical mapping system guidance and helps in reducing radiation exposure to patients and operators (14, 15). It is difficult to puncture through the atrial septum with abnormal anatomical structures, such as atrial septal thickening and aneurysm, lipoma, atrial septum after previous cardiac surgery, and implantation of atrial septal occlude (16). Sometimes, due to its inability to be located under conventional fluoroscopy, certain complications occur, namely, pericardial tamponade, aortic root puncture, arterial embolism, and pulmonary vein perforation (17). ICE usage is more advantageous in abnormal atrial septal puncture cases to avoid life-threatening complications and improve the success rate of the procedure. However, it should be noted that for patients with cardiac implantable electronic devices, special consideration should be given due to the risk of electrode shift caused by ICE (15).

### Application of intracardiac echocardiography in the ablation of outflow tract arrhythmias

Idiopathic outflow tract arrhythmias include premature ventricular complex (PVC), unsustained and sustained ventricular tachycardia (VT), respectively. The majority of outflow tract arrhythmias originate from a focal mechanism that includes enhanced automaticity, triggered activity, and micro-reentry and is unrelated to either scar formation or ion channels (1).

**FIGURE 5:**
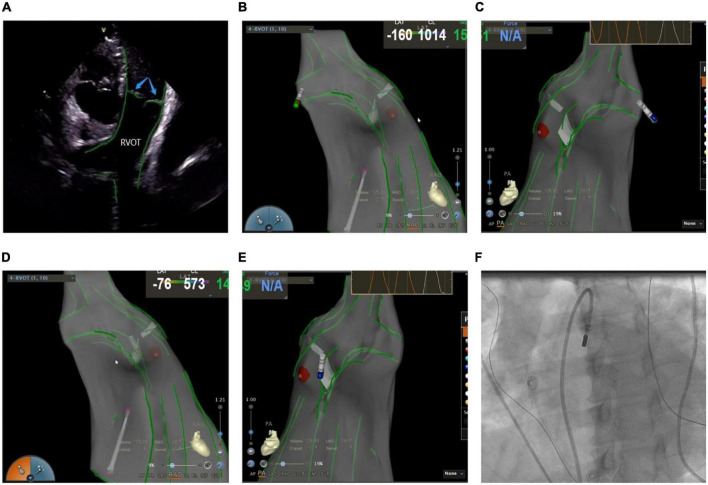
**(A)** Anatomic reconstruction of right ventricular outflow tract under ICE. RVOT: right ventricular outflow tract; Blue arrow: pulmonary valve. **(B,C)** Image of the catheter successfully entering the pulmonary artery under the CARTOSOUND module. **(B)** Right anterior oblique view; **(C)** pulmonary artery view. **(D,E)** Image of the catheter successfully attached to the pulmonary sinus in a reverse U-shape under the CARTOSOUND module. **(D)** Right anterior oblique view; **(E)** pulmonary artery view. **(F)** Successful placement of the catheter above the pulmonary sinus was verified by angiography.

Mostly idiopathic, right ventricular outflow tract arrhythmias are the most common form of clinical ventricular arrhythmias, accounting for about 80% of the total outflow tract ventricular arrhythmias (18). The myocardial tissue of the RVOT anatomically extends to the pulmonary valve and the pulmonary artery, which makes the ablation target location more complex. A previous study suggested that about 90% of the subjects had myocardial extension above the pulmonary valve, whereas nearly half (46%) of the RVOT arrhythmia lesions were located above the pulmonary valve (19). In recent years, inverted U-shaped ablation above the pulmonary valve has been widely popularized due to an increased understanding of the RVOT ablation mechanism (20, 21). ICE technique shows the precise position and relationship between the ablation catheter, pulmonary valve, pulmonary artery, and RVOT defines the position of supra-and subvalvular reflexes, visualizes the adhesion and stability of the ablation catheter and the target tissue in real-time as well as monitors the occurrence of procedural complications like valve perforation (Figure 5). Due to the complex anatomy surrounding RVOT, radiofrequency energy transmitted near coronary arteries can lead to the occlusion of major epicardial vessels like the left anterior descending coronary artery, thereby causing myocardial infarction in some cases (22). Hence, it’s critical to identify the functional status of the coronary arteries. De Sensi et al. (23) and Ho (24) reported several successful cases of inverted U-shaped RVOT under the guidance of ICE. The pulmonary artery, aortic valve, left anterior descending branch, and RVOT were precisely monitored in real-time, thus, avoiding the use of radiography and contrast agents, with no perioperative complications.

The incidence of LVOT arrhythmias, especially ventricular premature or ventricular tachycardia originating from the aortic sinus and its adjacent areas, is notably increasing year by year. The aortic sinus is located in the center of the heart, and its adjacent anatomical structure is complex, and the origin of some patients may be close to important anatomical structures (such as coronary arteries, etc.). Any injury to this area may lead to valve injury, myocardial perforation, or fatal complications like acute myocardial infarction and complete atrioventricular block, resulting in an increased risk of surgery. Accurate identification of the coronary ostium significantly reduces the risk of coronary artery injury while ablating the target area. Furthermore, the distance between the coronary ostium and the catheter tip > 1 cm is considered safe for ablation procedures (1, 2). Although angiography has been commonly used in the past, it suffers from many limitations when judging the position of catheters and arteries (25). Firstly, the relative distance between the artery and the catheter might vary according to the heart cycle and subtle catheter movements. Secondly, angiography cannot be performed continuously during an ablation procedure. And thirdly, the shape and size of the lesions might become risk factors for the injury that are not related to direct arterial contact. When angiography is compared with ICE in terms of functional accuracy, it is suggested that ICE provides high-resolution real-time visualization of the left main coronary artery ostium in relation to the short-axis section of the aortic sinus, whereas the right coronary artery ostium is seen when the ICE probe points above the right coronary sinus. Furthermore, when ICE guides the catheter’s position either at the bottom of the aortic sinus or the junction of the two cusps, the catheter is usually placed > 1 cm from the coronary orifice. Based on this, it can be stated that ICE can replace coronary angiography in evaluating the catheter’s stability and association with the adjacent structures like aorta, aortic valve, and coronary artery orifice by real-time monitoring, and thus, can provide safe surgical interventions as well as improve the procedural success rate.

**FIGURE 6:**
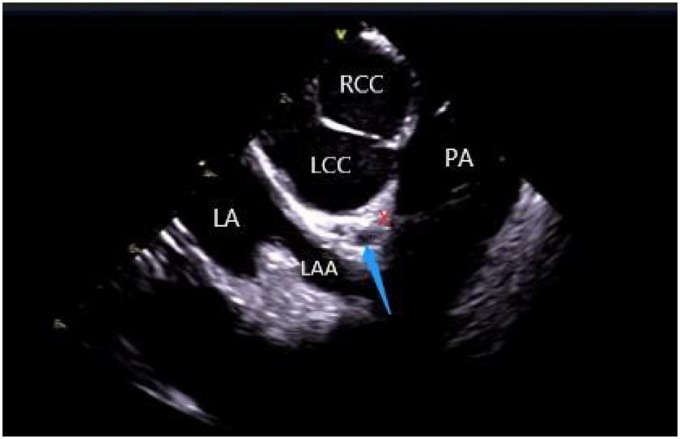
ICE view of the aortic root and pulmonary artery at the left ventricular summit region, viewed from the right ventricular outflow tract. At the center, a short axe of the left main coronary artery (LMCA) (blue arrow) surrounded by a thick layer of adipose tissue (LVS). RCC, right coronary cusp; LCC, left coronary cusp; LA, left atrial; LAA, left atrial appendage; PA, pulmonary artery; The LVS is marked with an X.

The left ventricular summit (LVS) is the triangular region at the most superior part of the LV epicardial surface consisting of the left circumflex coronary artery, the left anterior descending artery, and an approximate line from the first septal coronary artery laterally to the left AV groove. As the LVS region is bisected by the great cardiac vein (GCV), an area superior to it is inaccessible to catheter ablation due to the proximity of the coronary arteries and overlying epicardial fat (26). It is suggested that PVCs in this area sometimes require “anatomical ablation,” and the successful target may not be the earliest source of excitement (27). In the traditional 3D model, the presence of an unexplored area might result in incomplete model construction and ablation failure. Hence, coronary angiography or cardiac venography is usually used to guide the localization, while the distance between the ablation catheter and the epicardial coronary artery exceeding 0.5 cm is considered safe to avoid vascular damage (28). However, some studies have revealed that catheter ablation of adjacent structures like the aortic valve and RVOT under ICE-assisted imaging is highly effective (29) (Figure 6). Furthermore, although the selective resolution of the ICE prohibits the viewing of the small veins, the operator can successfully perform ablation in the branch without venography. Rivera et al. reported successful zero-ray ablation procedures in 26 cases of PVC/VT originating from the LVS region, with an immediate success rate of 84% due to the guidance of an ICE-guided 3D electroanatomical mapping system without any serious complications (30).

### Application of intracardiac echocardiography in ablation of arrhythmias originating from papillary muscles

Due to their unique anatomy, PMs are a source of ventricular arrhythmias (VAs) in both normal and abnormal cardiac structures (31). The left ventricular papillary muscle connects the mitral valve chordae tendineae to the LV with posteromedial and anterolateral papillary muscles. The left anterolateral papillary muscle, originating from the anterior LV wall, is connected to the anterior part of the two mitral lobes *via* chordae tendineae, while the left posteromedial papillary muscle, derived from the posterior inferior LV septum, gets attached *via* chordae tendineae to the middle and posterior parts of the two mitral lobes. Owing to the variable anatomy of LV papillary muscle, it can have single or multiple heads. On the contrary, the RV papillary muscle is divided into three portions: septal, posterior, and anterior papillary muscles that connect the RV myocardium to the TV *via* the tricuspid chordae tendineae (1). The septal papillary muscle is closely associated with parts of the right bundle branch (RBB).

**FIGURE 7:**
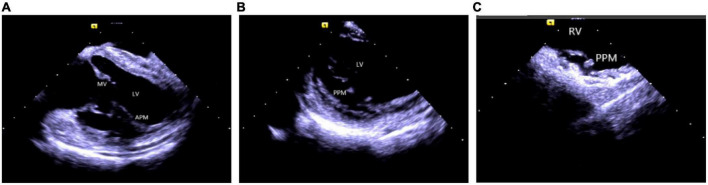
Papillary muscle. **(A)** Left ventricular anterior papillary muscle. **(B)** Left ventricular posterior papillary muscle. **(C)** Right ventricular posterior papillary muscle. MV, mitral valve; LV, left ventricle; APM, anterior papillary muscle; PPM, posterior papillary muscle; RV, right ventricle.

Catheter ablation is highly effective yet challenging because of complex PM anatomy, their independent movements during the cardiac cycles, the ambiguous origin of arrhythmias along with catheter-tissue contact instability. They were associated with a higher rate of local recurrence rate and had a lower success rate when compared with VA from other sites (32). Certain disadvantages of a 3D electroanatomical mapping system like the improper orientation of the catheter to the PM as well as operation of the catheter may sometimes lead to premature termination of arrhythmia and subsequent ablation failure (33). Integration of ICE with 3D electroanatomical mapping systems has further increased the efficacy of real-time monitoring of both catheter and local anatomy, along with accurate identification of the PMs structure (size, shape, and number of heads). An important factor governing a successful ablation is the appropriate contact between the ablation catheter and the targeted tissue, as well as the correct location and stability of the catheter tip for optimum outcome (1, 2, 34). A single-center study conducted on more than 100 people described that ICE effectively identifies the catheter’s location and lesion distribution segments, reduces the surgical time and radiation exposure, and thus, improves the overall success rate of surgery (35). Rivera et al. stated that CFS RF/ICE is the most effective way to reduce the consumption of antiarrhythmic drugs and dynamic ECG load after catheter ablation in LV papillary muscle origin arrhythmias (36). A strong correlation was also observed between the ICE usage and successful procedural outcomes, whereas the recurrence rate in patients undergoing ablation without ICE was 20 times higher than in ICE patients. Furthermore, ICE can duly identify abnormal PM echoes and the existence of scars, thus, recognizing the exact origin of arrhythmias (Figure 7).

### Application of intracardiac echocardiography in ablation of arrhythmias originating from bundle branch

Idiopathic left ventricular arrhythmia is derived from the left bundle branch and has three manifestations on body surface electrocardiogram (ECG): (1) left posterior fascicular (LPF) ventricular arrhythmia, whose QRS morphology exhibits a right bundle branch block (RBBB) configuration and a superior axis; (2) left anterior fascicular (LAF) ventricular arrhythmia, which displays an RBBB configuration and right-axis deviation; and (3) upper septal fascicular (USF) ventricular arrhythmia, with a narrow QRS configuration and normal or right-axis deviation. LPF ventricular arrhythmia is the most common type of fascicular ventricular arrhythmia and accounts for 90% of the cases (37). Since the left posterior branch VT should be correctly distinguished from the VT of papillary origin, due to their proximity in anatomical positions, the distinction between the two variants by ECG and cardiac electrophysiology becomes difficult (38). It has been shown that the mechanism of branched VT is closely related to the association between the Purkinje fibers and the FT distributed around the PMs. Since anatomical structures surrounding traditional left-median posterior septal regions like left posterior PM or the FT are in proximity during the onset of VT, they are difficult to distinguish under ordinary 3D measurement systems. ICE displays the association between ablation catheter and left ventricular septum’s positions, PM and FT during the surgery and clarifies the true anatomical position of the best target, along with the degree of the catheter and the target adhesion. Consequently, it plays a highly significant role in exploring the mechanism of left posterior branch VT and improving the surgical success rate (33, 39).

### Application of intracardiac echocardiography in ablation of arrhythmias originating from moderator band

**FIGURE 8:**
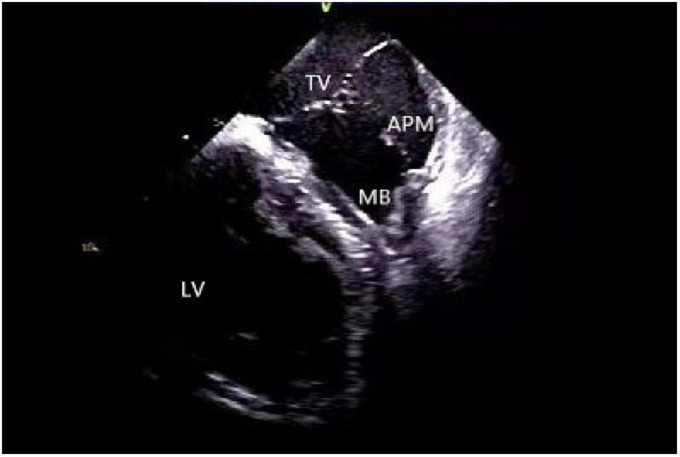
Moderator band. TV, tricuspid valve; LV, left ventricle; APM, anterior papillary muscle; MB, moderator band.

A moderator band is a muscular band located in the mid to apical RV that connects the interventricular septum to the RV-free wall, supporting the anterior papillary muscle. It typically contains an RBB subdivision and is one of the possible origin sites for arrhythmias (31, 40). Using a 3D mapping system alone for the ablation of moderator band arrhythmias is not highly effective due to uncontrollable stability as well as mapping and ablation catheter arrival rate. ICE is particularly useful in such cases as it clearly shows the anatomical structures adjacent to the moderator band, which is helpful for mapping and ablation. Furthermore, the construction of the long and short-axis views of the moderator band can clearly define the position of the band body, the septal and the anterior papillary muscle insertion points, and monitor the attachment and stability of the catheter and the target tissue in real-time (11, 41) (Figure 8).

### Application of intracardiac echocardiography in ablation of arrhythmia originating from false tendon

A false tendon is a common intraventricular anatomical variation (42), characterized by a fibrous or fibromuscular chord-like band that crosses the LV cavity and gets attached to the septum, PMs, trabeculations, or the LV free wall. Due to the presence of conductive tissue, some ventricular arrhythmias may originate from this site (42–44). The existence of FT hinders the operation of intracardiac catheterization while its complex anatomy, independent movement during the cardiac cycle, and unstable catheter-tissue contact pose challenges to mapping and ablation. Using ICE effectively improves the safety and feasibility of surgical technique by constructing an LV anatomical model, describing the relative relationship between anterior and posterior papillary muscles, free wall, and FT, completing target mapping in direct vision, and real-time monitoring of the catheter’s stability and ablation structures (45, 46).

### Application of intracardiac echocardiography in ablation of ventricular arrhythmias originating in the vicinity of the his bundle

**FIGURE 9:**
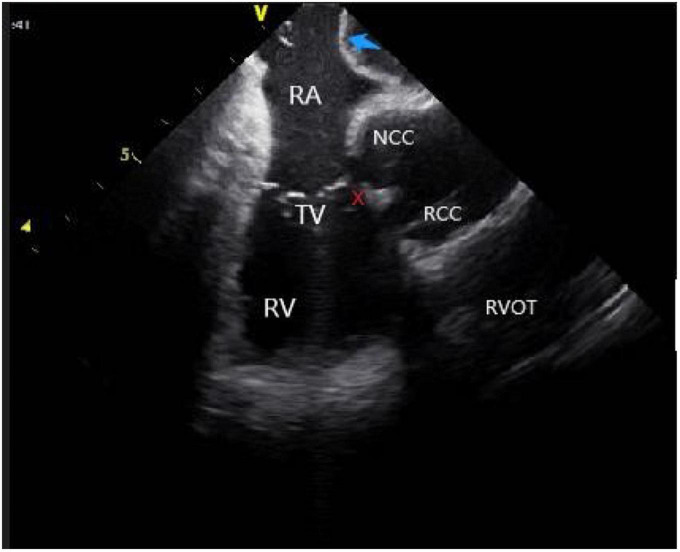
The anatomy of para-his area under ICE. RV, right ventricular; RA, right atrial; NCC, non-coronary cusp; RCC, right coronary cusp; TV, tricuspid valve; RVOT, right ventricular outflow tract; Blue arrow, atrial septum; The His region is marked with an X.

VT and PVC originating in the vicinity of the His bundle account for 3–9% of all idiopathic Vas (47). For arrhythmias originating in this area, it is necessary to map their adjacent structures in detail because of their complex anatomy structure (48). ICE constructs the left and right His bundle area, RA, RV, tricuspid annulus, outflow tract, and interatrial septum in detail, which becomes convenient for the operator to understand the local anatomy (Figure 9). Under direct ultrasound vision, the operator can determine the earliest activation time and achieve a higher success rate by utilizing activation maps along with cardiac pacing and mapping, avoiding the conduction area and reducing the possibility of cardiac block. If the earliest ventricular activation is observed near the RV His bundle area, the ablation target distance from the maximum His potential should be > 5 mm for the safety and feasibility of the operation (48).

Tricuspid annulus refers to the area immediately adjacent to the TV, including the septal, free wall, and para-His regions. In general, the septal region can be divided into three areas: the anterior, middle, and posterior septa. The bundle of His passes through the anterior septal region; a majority of idiopathic VAs in its vicinity originate from the anterior and middle septal areas. The application of ICE makes catheter ablation safe and more feasible. Under the direct vision of ICE, the C-shaped curve of the ablation catheter is reversed under the TV septal lobe by using the adjustable curved long sheath to make the ablation catheter stable while protecting the atrioventricular node from radiofrequency energy damage (49, 50).

### Identification and ablation of scar (patch)

**FIGURE 10:**
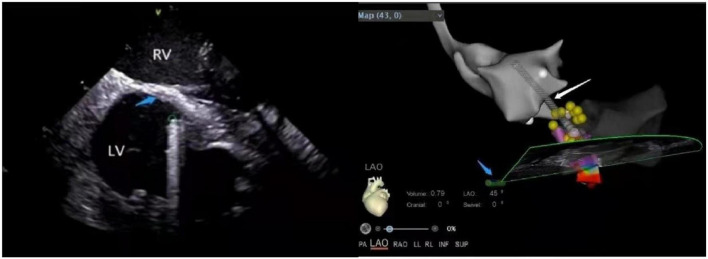
**(Left panel)** The ventricular septal scar under ICE. LV, left ventricular; RV, right ventricular; Green circle, the position of the ablation catheter tip; Blue arrow, scar (increased echo density). **(Right panel)** The position of the ablation catheter tip under the CARTOSOUND module in the ablation of the scar. Left ventricular outflow tract (gray); Conduction beam (yellow balls); White arrow, ablation catheter; Blue arrow, ICE catheter.

In patients who have undergone LV reconstruction, the arrhythmias mostly originate in the scar or patch boundary (51) or the myocardium below the patch. When the origin is in the myocardium below the patch, most of them need to be ablated *via* an epicardial pathway. However, a study stated that ICE-guided catheter ablation of an arrhythmogenic substrate under the patch *via* an endocardial approach is safe and feasible (52). ICE also identifies and depicts the scars of the endocardium, mid-myocardium, and epicardium, defines the local anatomy, and evaluates the distribution of arrhythmogenic substrate (9) (Figure 10). In ischemic cardiomyopathy, the scar is visualized as an area of myocardial hypokinesia/akinesia associated with thinning and hyper-echogenicity and corresponds to a coronary territory displaying voltage and electrogram abnormalities on electroanatomic mapping. In non-ischemic cardiomyopathy, ICE also detects the presence of mid-myocardial and epicardial scar (8), which correlates with unipolar endo-and epicardial bipolar voltage abnormalities. Furthermore, for epicardial ventricular tachycardia, ICE-guided pericardial puncture and catheter ablation exhibits better safety. ICE enables real-time visualization of the puncture, minimizing the risk of inadvertent RV puncture, cardiac tamponade, and pericardial bleeding. In addition, during epicardial mapping and ablation, the catheter tip was tagged and located on ICE imaging to ensure stable attachment of the catheter to the epicardial scar. Finally, ICE defines the specific reference point of the aneurysm neck by describing the inflection point between the aneurysm and the normal tissue (53). Because of the large area of myocardial thinning in the aneurysm, continuous ICE catheter monitoring is essential to avoid myocardial perforation during mapping and ablation.

### Other applications of intracardiac echocardiography in ablation

Inaba et al. proposed a contraction mapping method in which the ICE probe located in the right cardiac system defined the location of early myocardial contraction required for detailed mapping while excluding the location of myocardial delayed contraction, which was insignificant (54). This method helped limit the activation range and cardiac pacing mapping and reduced the surgical time. This mapping method might work well in cases of mapped VAs originating from the left side due to the predictable local activation time for the LV without any transseptal or arterial puncture.

The posterior-superior process of the LV is the most inferior and posterior aspect of the basal LV, extending posteriorly to the plane of the TV. Since the LV posterior-superior process is anatomically adjacent to the medial and inferior side of the right atrium, the arrhythmia originating from the LV posterior-superior process can successfully be ablated from the RA (48). Santangeli et al. reported a successful case of ICE-guided ablation of VAs arising from the left posterior-superior process *via* the right atrial approach (55). The best view of the inferior medial RA and adjacent LV can be obtained by clockwise rotation and anterior deflection of the catheter in the HomeView, where it is placed on the inferior and medial side of the RA, opposite to the earliest activation position of the left ventricular endocardium. Hence, ICE is essential for determining the anatomical relationship between the left ventricular posterior-superior process and the adjacent RA and visualizing the tissue contact and stability in real-time during ablation.

## Monitoring of complications

ICE plays an important role in identifying and monitoring surgical complications. Real-time evaluation of the heart by ICE helps operators in evaluating the possible causes of complications and taking corrective measures to minimize the adverse consequences. The role of ICE in the early identification of complications has been listed as a Class I recommendation in the 2019 HRS/EHRA/APHRS/LAHRS expert consensus statement on Catheter Ablation of Vas (1).

**FIGURE 11:**
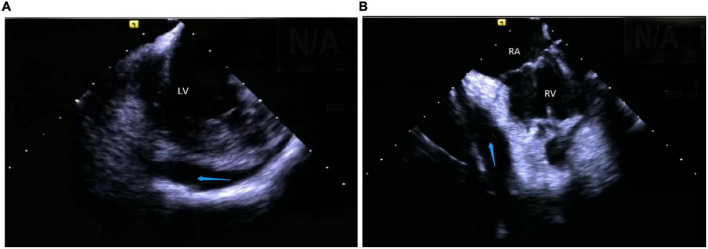
Still frame of pericardial effusion occurring during a catheter intervention under ICE. **(A)** Left ventricular pericardial effusion. **(B)** Right ventricular pericardial effusion. LV, left ventricular; RV, right ventricular; RA, right atrial; Blue arrow, the positions of the pericardial effusion.

Pericardial effusion and cardiac tamponade are the most serious complications occurring in the process of ablation, which occur rapidly when puncturing the cardiac structures, such as the atrial septum, left atrial appendage, etc. ICE monitors pericardial fluid accumulation along with atrial and ventricular compression caused by pericardial tamponade in real-time, detects pericardial effusion before hemodynamic changes, and implements early interventions (3–5) (Figure 11 and Supplementary Video 1). Additionally, ICE also evaluates the severity of pericardial effusion in patients with intraoperative hemodynamic deterioration (56). Notably, intracardiac echocardiographic differentiation of fat from fluid could be subtle. Several clues can be useful to distinguish epicardial fat from pericardial effusion. First, ICE can detect invagination of the right atrial or ventricular wall during diastole and early systole as an important sign of pericardial effusion and even cardiac tamponade (Supplementary Video 1). Second, fat is usually located more anterior than posterior. Third, fat is slightly less mobile and pericardial layers move less freely. Fourth, fat is usually slightly more echogenic or granular than fluid.

Cardiogenic shock, characterized by a progressive decrease in blood pressure during the surgery or inability to maintain blood pressure after electrical cardioversion, is more common in patients with left ventricular dysfunction. The resultant ICE imaging shows a stagnation of cardiac activity (2), which requires urgent treatment.

**FIGURE 12:**
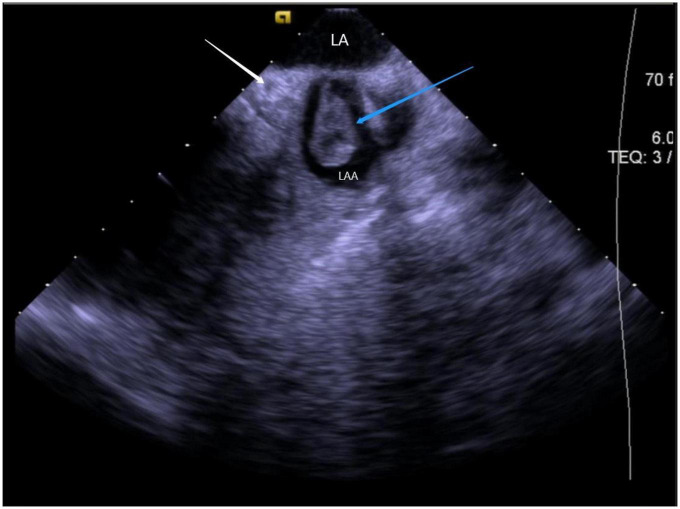
Still frame of formation of thrombus under ICE. LA, left atrial; LAA, left atrial appendage; Blue arrow, thrombus in the left atrial appendage; White arrow, LCX (left circumflex artery).

Since ICE also detects the formation of intracardiac (6), the sheath tip, and catheter thrombi, thus, potential therapeutic interventions can be taken before their occurrence (7). ICE imaging displays thrombi as echo-dense reflecting masses with defined margins that are distinct from the underlying endocardium and observed in multiple imaging planes without any relation to pectinate muscles, FT, or trabeculae (57) (Figure 12). The appearance of spontaneous echo contrast due to low-flow conditions preceding the thrombus formation is also assessed (4). Once ICE detects a soft thrombus, the clot can be sucked into the sheath tube, and higher doses of anticoagulants can be administered to prevent serious thromboembolic complications (58). Sometimes, ICE can guide the withdrawal of the thrombus to the RA if the thrombus is firmly attached to the catheter (59).

**FIGURE 13:**
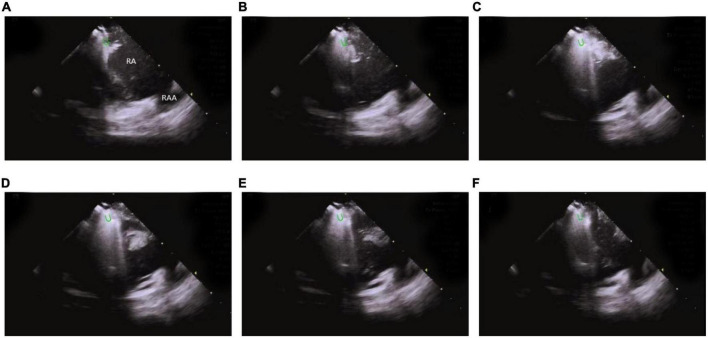
Steam-pop formation during RF **(A–F)**. **(A,B)** The microbubbles were observed before steam pop; **(C–F)** ICE imaging showed that the formation suddenly expanded to a sphere over the course of several seconds. Green semi-ellipse: the positions of the ablation catheter tip; RA, right atrial; RV, right ventricle; RAA, right atrial appendage.

ICE monitors the stability of catheter-tissue contact and the formation of lesions, thus, providing operators with key information to avoid steam popping and myocardial perforations. Any other signs of excessive tissue temperature, such as a local increase in cardiac echoes (excessive whitening of the catheter tip and adjacent cardiac tissue) or a sudden increase of microbubbles (signs before steam popping), can also be successfully detected (60) (Figure 13 and Supplementary Video 2).

This technique also keeps track of valve changes before, during, and after the surgery, along with complications such as valvular regurgitation caused by valvular insufficiency and rupture of chordae tendineae.

## Limitations of intracardiac echocardiography

Firstly, ICE has limited application in terms of spatial accuracy and resolution. Although ICE can reveal PM, FT, and other protruding or suspended structures in the cardiac cavity, it seldom achieves accurate imaging for a few smaller anatomical structures, such as fibromuscular bands and trabeculae carneae (33). Secondly, although ICE plays an important role in early intraoperative monitoring of complications, its ability to reduce the incidence of complications is still uncertain. Some studies have shown that ICE can reduce the incidence of complications during pulmonary vein isolation (61), but in the ablation of VAs, there is no comparative study recommended in the 2019 expert consensus statement to clarify the relationship between them. Several studies have suggested that ICE minimizes the incidence of complications by identifying relevant anatomical structures and real-time catheter localization (4, 62–64). Thirdly, the operating space of the ICE catheter is limited due to the restricted size of the cardiac cavity. Often, multiple mapping catheters are placed in the cardiac cavity, which reduces their functional capacity and maneuverability. Fourthly, as ICE usage requires systematic echocardiographic training for interventional physicians, the proficiency of interventional physicians in operating ICE might affect the safety and effectiveness of the surgery (8, 65, 66). Lastly, the price of ICE ultrasound catheters is high, and as disposable catheters are commonly used, their utilization is limited to some extent.

## Summary and future perspectives

ICE allows real-time visualization of the mapping and ablation of ventricular arrhythmias and dynamically displays the relationship between the catheter and specific anatomical structures. It also plays an important role in maintaining the catheter tip-tissue contact and attachment, monitoring the formation of lesions, early identification of surgical complications, and reducing fluoroscopy time. Further improvements in existing healthcare models like increased imaging quality, appropriate catheter size, 3D imaging capability, and cost-effectiveness will make ICE a more widely used treatment modality in the ablation of ventricular arrhythmias.

## Author contributions

CM, TC, YC, JG, WH, and QW outlined, drafted, and contributed to the writing of the manuscript. JZ contributed to the review of the manuscript. All authors listed have contributed sufficiently to the project in order to be included as authors and approved the final version of the manuscript for publication.
